# Predictive models of long‐term survival outcomes following radical cystectomy

**DOI:** 10.1002/cam4.6670

**Published:** 2023-10-30

**Authors:** Akira Ohtsu, Seiji Arai, Yuji Fujizuka, Yoshiyuki Miyazawa, Masashi Nomura, Yoshitaka Sekine, Hidekazu Koike, Hiroshi Matsui, Yasuhiro Shibata, Kazuto Ito, Kazuhiro Suzuki

**Affiliations:** ^1^ Department of Urology Gunma University Graduate School of Medicine Maebashi Japan; ^2^ Department of Urology Takasaki General Medical Center Takasaki Japan; ^3^ Department of Urology Kurosawa Hospital Takasaki Japan

**Keywords:** bladder cancer, cystectomy, GUOSG score, predictive model

## Abstract

**Background:**

Identifying the likelihood of life‐threatening recurrence after radical cystectomy by reliable and user‐friendly predictive models remains an unmet need in the clinical management of invasive bladder cancer.

**Methods:**

A total of 204 consecutive patients undergoing open radical cystectomy (ORC) for bladder cancer were retrospectively enrolled between May 2005 and August 2020. Clinicopathological and peri‐ORC therapeutic data were extracted from clinical records. We explored predictive factors that significantly affected the primary endpoint of overall survival (OS) and secondary endpoints of cancer‐specific survival (CSS) and recurrence‐free survival (RFS).

**Results:**

During a median follow‐up of 3.9 years, 42 (20.6%) and 10 (4.9%) patients died due to bladder cancer and other causes, respectively. Five‐year RFS, CSS, and OS were 66.5%, 77.6%, and 75.4%, respectively. Pathological T and N categories and lymphovascular invasion (LVI) significantly affected RFS by Cox regression analysis. Accordingly, clinical T and pathological N categories and LVI significantly affected CSS. Clinical T and pathological N categories, LVI, age, and ORC tumor grade significantly affected OS. Based on the assessment score for each independent risk factor, we developed the Gunma University Oncology Study Group (GUOSG) score, which predicts RFS, CSS, and OS. The GUOSG score classified four groups for RFS, three for CSS, and five for OS, with statistically significant distribution for nearly all comparisons.

**Conclusions:**

The GUOSG model is helpful to show individualized prognosis and functions as a risk‐stratified historical cohort for assessing the lifelong efficacy of new salvage treatment regimens.

## 1 INTRODUCTION

Bladder cancer is the 10th most common cancer worldwide, and incidence and mortality were estimated at 573,743 (age‐standardized incidence rate: 5.6/100,000) and 212,536 (age‐standardized mortality rate: 1.9/100,000) in 2020, respectively.^1^ In Japan, the estimated incidence was 23,383 in 2019,^2^ while mortality was 9443 in 2021.^3^ Radical cystectomy and pelvic lymph node (LN) dissection, in combination with or without neoadjuvant/adjuvant chemotherapy, is the standard of care for patients with locally advanced or intractable bladder cancer with muscle invasion, multiple G3 pT1, or Bacille de Calmette et Guérin (BCG) refractory carcinoma in situ (CIS). However, the curative ability of currently available multimodal treatments has not met expectations, and survival outcomes after radical cystectomy vary even if clinicopathological features seem identical. For instance, clinical T2N0 bladder cancer can be treatable with radical cystectomy, whereas the survival time varies among individuals. Therefore, assessing objective clinicopathological risk factors is a high priority in patients who may progress to life‐threatening recurrence after radical cystectomy. A well‐known prognostic model to estimate the likelihood of disease‐specific death is the COBRA score, which requires information regarding age, tumor stage, and LN density.^4^ In the present study, we investigated the impacts of almost all available pre‐, intra‐, and post‐open radical cystectomy (ORC) clinicopathological and treatment‐related factors on overall survival (OS), cancer‐specific survival (CSS), and recurrence‐free survival (RFS), using consecutive ORC series treated at our institution, and created a reliable and user‐friendly predictive model specific for each survival outcome.

## 2 PATIENTS AND METHODS

A total of 204 consecutive patients undergoing ORC for bladder cancer at a single institution between May 2005 and August 2020 were enrolled in the present retrospective registry study. We extracted the clinical records for pre‐/intra‐/post‐ORC clinicopathological data and the presence and regimens of systematic chemotherapy before and after ORC. Pre‐ORC data included age, sex, body mass index (BMI), serum hemoglobin, presence of hydronephrosis, tumor histological type and grade, presence of CIS, clinical TNM stage (TNM classification 8th edition, UICC: 2017) at transurethral resection of bladder tumor, time from muscle‐invasive or intractable BCG refractory bladder cancer diagnosis to either neoadjuvant chemotherapy (NAC) or ORC, and regimen of NAC used. Intra‐ORC data included surgery time, blood loss during surgery, allogenic blood transfusion, and upper urinary tract involvement at rapid pathology during surgery. Post‐ORC clinicopathological data contained the presence and regimen of adjuvant chemotherapy, pathological TNM stage, tumor grade, lymphovascular invasion (LVI), CIS, surgical margin, downstaging to pT0, and upstaging at ORC. A clinical conference at our department judged the decision to undergo NAC and adjuvant chemotherapy. Specifically, we vigorously recommended NAC for patients with clinically positive LN or locally advanced (>cT3) bladder cancer and adjuvant chemotherapy for patients with aggressive clinicopathological features in ORC specimens. Patients with estimated glomerular filtration rate (eGFR) >60 tended to receive cisplatin‐based chemotherapy, and other patients (eGFR <60) either received carboplatin‐based chemotherapy or did not receive NAC.

Patients were followed up at least every 2 months for the first 2 years after surgery, every 3 months for the next 3 years, semiannually for 10 years, and annually thereafter. Follow‐up examinations to check for disease recurrence were blood tests including tumor markers (CA 19‐9 for urothelial carcinoma, SCC for squamous cell carcinoma) or urine cytology, alternately, at each visit, and chest to pelvic computed tomography (CT) every 6 months for 3 years, annually for 5 years, and then every 1–2 years for 10 years.

The primary endpoint was OS, which was time from the date of ORC to any cause of death. The secondary endpoints were CSS, which was time from the date of ORC to cancer‐specific death, and RFS, which was time from the date of ORC to any clinical recurrence (local recurrence in the surgical field, intra‐urinary recurrence, LN, or distant metastasis), or any cause of death.

All statistical analyses were performed using EZR (Saitama Medical Center, Jichi Medical University, Saitama, Japan), a graphical user interface for R (The R Foundation for Statistical Computing, Vienna, Austria). More precisely, it is a modified version of R commander designed to add statistical functions frequently used in biostatistics. The Kaplan–Meier method was used to estimate all survival outcomes. Cutoffs of the above clinicopathological factors for Kaplan–Meier analyses were explored by separating patients into binary, tertiary, or quaternary models for continuous variables or into two to seven groups for categorized variables to establish more significant and meticulous separation. If two adjacent subgroups were considered to have an equal predictive value, they were combined into one subgroup. Significance among explored subgroups for each survival outcome was determined using log‐rank tests. The Cox proportional hazards model was used to determine independent significant predictive factors. The forward‐backward stepwise selection method was used to analyze independent surrogate factors to predict each survival outcome. A *p* value of <0.05 was considered statistically significant. Independent predictive factors for each survival outcome finally selected by the above stepwise multiple regression analysis were scored +1 in each log10 HR increased +0.5. The Gunma University Oncology Study Group (GUOSG) predictive models for RFS, CSS, and OS were established using corresponding assessment scores.

## 3 RESULTS

Clinicopathological background before and after ORC is shown in Table 1. Of the 204 patients, 56 (27.5%) underwent NAC before ORC, and 48 (23.5%) underwent adjuvant chemotherapy after ORC. NAC regimens were GC or GCarbo in 48 patients (except for both regimens in one patient, +TS‐1 regimen in two patients), gemcitabine in one patient, GCarbo + TGCarbo (paclitaxel, gemcitabine, and carboplatin) in one patient, and M‐VAC in six patients (+GC regimen in two patients). Adjuvant chemotherapy regimens were mainly GC or GCarbo in 44 patients. Patients with clinical node positivity in preoperative CT and those with aggressive clinicopathological features in ORC specimens tended to undergo NAC and adjuvant chemotherapy, respectively (Table S1).

**TABLE 1:** Clinicopathological background of bladder cancer patients undergoing open radical cystectomy at Gunma University.

No. of patients	204	
Age at ORC, years, median (range)	70	(32–87)
Sex, *n* (%)		
Male	163	(79.9)
Female	41	(20.1)
BMI (median, kg/m^2^)	21.9	
Preoperative Hb (mean, g/dl)	11.7	
Hydronephrosis, *n* (%)		
Yes	45	(22.1)
No	159	(77.9)
Tumor histology at TUR‐BT, *n*, (%)		
UC	180	(88.2)
SCC	4	(2.0)
Adenocarcinoma	3	(1.5)
Others	17	(8.3)
Clinical T category, *n* (%)		
T1	58	(28.4)
T2	116	(56.9)
T3/T4a	30	(14.7)
Clinical nodal stage, *n* (%)		
N0	172	(84.3)
N1	25	(12.3)
N2, N3	7	(3.4)
TNM stage (TNM 8th edition), *n* (%)		
0isI (BCG refractory, etc.)	51	(25.0)
II	99	(48.5)
IIIA (N0)	22	(10.8)
IIIA (N1)	25	(12.3)
IIIB	7	(3.4)
IVA	0	(0.0)
Tumor grade at TUR‐BT, *n* (%)		
G1	1	(0.5)
G2	71	(34.8)
G3	132	(64.7)
CIS at TURBT, *n* (%)		
Yes	43	(21.1)
No	161	(78.9)
Time to treatment^a^, months, median (IQR)	1.4	(0.9–1.9)
Neoadjuvant chemotherapy (NAC), *n* (%)		
Yes	56	(27.5)
No	148	(72.5)
Treatment after ORC, *n* (%)		
Adjuvant chemotherapy		
None	156	(76.5)
Cisplatin or carboplatin‐based	44	(21.6)
Non‐cisplatin or non‐carboplatin‐based^b^	4	(2.0)

Abbreviations: CIS, carcinoma in situ; ORC, open radical cystectomy; TURBT, transurethral resection of bladder tumor.

^a^ Time to treatment was defined as time from muscle‐invasive bladder cancer or intractable BCG refractory bladder cancer diagnosis to either upfront RC or NAC.

^b^ Non‐cisplatin or non‐carboplatin‐based adjuvant chemotherapy: UFT (*n* = 3), TS‐1 (*n* = 1).

Intraoperative clinical data and pathological findings in the ORC specimens are shown in Tables 2 and 3. The prevalence of pathological LN metastases in lymph node dissection specimens at the common, external, and internal iliac and obturator fossa LN was 22.1% (45 patients). Dissected LN and positive LN numbers were recorded in 43 patients (four with positive LNs) since 2017, and these median values were 14 (range, 12–16) and 0 (range, 0–1), respectively. Due to multiple missing values, we excluded these LN numbers for further analysis. Patients undergoing NAC tended to have downstaging of ORC specimens, and there was no significant correlation between the absence of NAC and upstaging of ORC specimens (Table S2).

**TABLE 2:** Intraoperative clinical data.

No. of patients	204	
Operation time, minutes, median (range)	293.5	(103–621)
Blood loss during operation, mL, median (range)	1034	(114–10,000)
Allogenic blood transfusion, *n* (%)	100	(49)
Upper urinary tract involvement at rapid pathology during operation		
Yes	40	
No	164	

**TABLE 3:** Pathological findings in radical cystectomy specimen.

No. of patients	204	
Pathological T category, *n* (%)		
pT0	28	(13.7)
pTa	4	(2.0)
pTis	27	(13.2)
pT1	33	(16.2)
pT2	38	(18.6)
pT3	55	(27.0)
pT4	19	(9.3)
Pathological N category, *n* (%)		
pN0	158	(77.5)
pN1	24	(11.8)
pN2	16	(7.8)
pN3	5	(2.5)
pNX	1	(0.5)
Tumor grade at RC		
No tumor	28	(13.7)
G1	3	(1.5)
G2	70	(34.3)
G3/variant histology	103	(50.5)
Lymphovascular invasion (LVI), *n* (%)		
Yes	75	(36.8)
No	129	(63.2)
CIS at ORC specimen, *n* (%)		
Yes	41	(20.1)
No	163	(79.9)
Surgical margin, *n* (%)		
None	198	(97.1)
Focal ureteric	6	(2.9)
Downstaging to pT0pN0 at ORC, *n* (%)		
Yes	27	(13.2)
No	177	(86.8)
Upstaging at ORC, *n* (%)		
No	170	(83.3)
Yes	34	(16.7)

Abbreviations: CIS, carcinoma in situ; LVI, lymphovascular invasion; ORC, open radical cystectomy.

At the time of the analyses (data cutoff, August 17, 2020), the median follow‐up time for surviving patients from the date of ORC was 3.9 years (range, 0.01–14.9 years). The 5‐year RFS, CSS, and OS rates were 66.5%, 77.6%, and 75.4%, respectively. Of 204 patients, 42 (20.6%) and 10 (4.9%) died due to bladder cancer progression and other causes, respectively. Metastatic progression of regional LN or distant sites after ORC was apparent in 61 patients (29.9%), with 15 (26.8% of 56) treated with NAC and 46 (31.1% of 148) without NAC (Table S3). LN and bone metastases were the most common in patients treated with NAC, whereas LN and lung metastases were the most common in the non‐NAC group.

RFS, CSS, and OS were estimated by Kaplan–Meier analysis and stratified by various preoperative and postoperative clinicopathological findings (Table S4). The prognostic impacts of each clinicopathological factor in the univariate analysis were almost identical in these three survival outcomes except age, which only affected OS. Clinical T (T3/4), pathological T (pT3/4), tumor grade (G2 + G3/variant histology), presence of LVI, absence of downstaging to pT0pN0 and presence of upstaging at ORC, and receipt of adjuvant chemotherapy resulted in significantly worse RFS, CSS, and OS. Pathological T category (pTis) was associated with significantly worse CSS than pT0 + pTa, while pathological N category was correlated with significantly worse RFS, CSS, and OS, except N1 versus N2 + 3 for CSS and OS.

Table 4 shows the final multivariate Cox regression model and risk assessment scores for RFS, CSS, and OS in 204 patients undergoing ORC. To explore independent clinicopathological factors and best cutoffs, which ideally predict RFS, CSS, and OS, all clinicopathological factors that significantly affected each survival outcome in univariate analyses were investigated in the full model for multivariate analyses. Clinicopathological factors were then restricted to the number of candidate prognostic factors ranked in the order of *p* value. We selected three factors independently affecting RFS and CSS and five factors affecting OS, concomitant with the corresponding best cutoffs. Log HR of approximately 0.5 was associated with 1 point in the assessment score, and all risk assessment scores in the groups affecting worse survival outcomes were scored as 1.

**TABLE 4:** Multivariate Cox regression model and risk assessment score for recurrence‐free survival (RFS), cancer‐specific survival (CSS), and overall survival (OS) in 204 patients undergoing radical cystectomy.

	*n*	Hazard ratio	95% CI	*p* Value	log HR	risk assessment score
RFS						
Pathological T category						
pT0 + pTa + pTis+pT1 + pT2	130	Reference				0
pT3 + pT4	74	1.869	1.0490–3.331	0.003391	0.27	1
Pathological N category						
pN0 + pN1	182	Reference				0
pN2 + pN3	21	2.665	1.4620–4.856	0.001367	0.43	1
LVI						
No	129	Reference				0
Yes	75	2.494	1.4060–4.424	0.001778	0.40	1
CSS						
Clinical T category						
T1 + T2	174	Reference				0
T3/4	30	2.219	1.111–4.434	0.02401	0.35	1
Pathological N category						
pN0 + pN1	182	Reference				0
pN2 + pN3	21	2.897	1.411–5.946	0.003751	0.46	1
LVI						
No	129	Reference				0
Yes	75	2.889	1.442–5.786	0.002756	0.46	1
OS						
Age, years						
<70	102	Reference				0
≥70	102	2.393	1.347–4.250	0.002906	0.38	1
Clinical T category						
T1 + T2	174	Reference				0
T3/4	30	2.304	1.2550–4.230	0.007095	0.36	1
Pathological N category						
pN0 + pN1	182	Reference				0
pN2 + pN3	21	2.594	1.284–5.240	0.007907	0.41	1
Tumor grade at ORC						
No tumor + G1	31	Reference				0
G2 + G3 + variant histology	173	3.758	0.8742–16.150	0.00752	0.57	1
LVI						
No	129	Reference				0
Yes	75	2.106	1.132–3.916	0.01865	0.32	1

*Note*: Log10 HR = 0.5; assessment score +1.

Abbreviations: CSS, cancer‐specific survival; HR, hazard ratio; LVI, lymphovascular invasion; ORC, open radical cystectomy; OS, overall survival; RFS, recurrence‐free survival.

**FIGURE 1:**
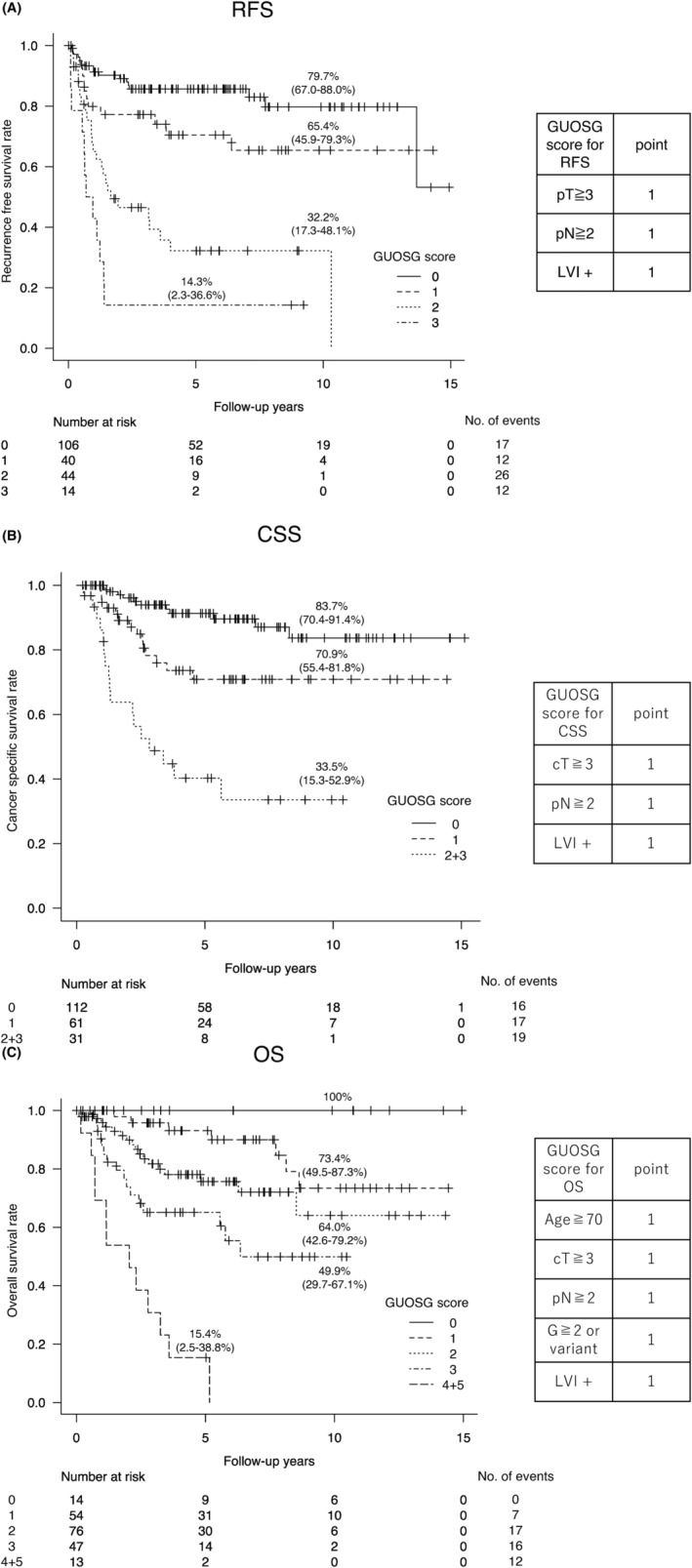
Survival outcomes stratified by Gunma University Oncology Study Group (GUOSG) score. (A) recurrence‐free survival, (B) cancer‐specific survival, and (C) overall survival.

The GUOSG score ranged from 0 to 3 and 0 to 5 for the RFS/CSS and OS models, respectively. Finally, the GUOSG scores were reclassified into four (0, 1, 2, 3), three (0, 1, ≥2), and five (0, 1, 2, 3, ≥4) groups in the models predicting RFS, CSS, and OS, respectively (Table 5 and Figure 1). RFS significantly differed between groups, except for the GUOSG score 0 versus 1 (*p* = 0.075). CSS was significantly different between any groups. OS significantly differed between the groups, except for GUOSG score 0 versus 1 (*p* = 0.112) and 1 versus 2 (*p* = 0.067).

**TABLE 5:** recurrence‐free survival (RFS), cancer‐specific survival (CSS), and overall survival (OS) stratified by Gunma University Oncology Study Group (GUOSG) score.

GUOSG score	*n*	Five‐year survival	95% CI	Ten‐year survival	95% CI	Median survival (months)	Log‐rank test *p* value
RFS							
0	106	0.857	0.770–0.913	0.797	0.670–0.880	NA	Score0 vs. score1 = 0.0748 Score0 vs. score2 = 1.57e‐10 Score0 vs. score3 = 5.2e‐13 Score1 vs. score2 = 0.00174 Score1 vs. score3 = 0.0000493 Score2 vs. score3 = 0.0314
1	40	0.704	0.526–0.826	0.654	0.479–0.793	NA	
2	44	0.322	0.173–0.481	0.322	0.173–0.481	20.3	
3	14	0.143	0.023–0.366	NA	NA	10.1	
CSS							
0	112	0.913	0.832–0.956	0.837	0.704–0.914	NA	Score0 vs. score1 = 0.00963 Score0 vs. score2 + 3 = 2.87e‐11 Score1 vs. score2 + 3 = 0.000825
1	61	0.709	0.554–0.818	0.709	0.554–0.818	NA	
2 + 3	31	0.402	0.217–0.581	0.335	0.153–0.529	34.5	
OS							
0	14	1.000	NA‐NA	1.000	NA‐NA	NA	Score0 vs. score1 = 0.112 Score0 vs. score2 = 0.0349 Score0 vs. score3 = 0.0055 Score0 vs. score4 + 5 = 0.000000861 Score1 vs. score2 = 0.0674 Score1 vs. score3 = 0.000769 Score1 vs. score4 + 5 = 3.58e‐15 Score2 vs. score3 = 0.05 Score2 vs. score4 + 5 = 0.00000000304 Score3 vs. score4 + 5 = 0.000264
1	54	0.931	0.799–0.977	0.734	0.495–0.873	NA	
2	76	0.757	0.626–0.847	0.640	0.426–0.792	NA	
3	47	0.651	0.474–0.782	0.499	0.297–0.671	77.3	
4 + 5	13	0.154	0.025–0.388	NA	NA	24.9	

Abbreviations: CI, confidence interval; CSS, cancer‐specific survival; OS, overall survival; RFS, recurrence‐free survival.

## 4 DISCUSSION

Invasive bladder cancer or BCG refractory CIS can be life‐threatening, even if treated with radical cystectomy with NAC or adjuvant chemotherapy. However, oncological outcomes vary mainly according to the tumor's aggressive features and partially due to the timing of diagnosis and appropriate effective chemotherapy regimens administered before and after radical cystectomy. Identifying a single risk factor that independently affects survival outcomes is essential. Significantly, several studies demonstrated the impact of a single clinicopathological factor (e.g., LVI,^5^, ^6^, ^7^ LN density,^4^, ^8^ pT stage,^7^, ^9^, ^10^, ^11^, ^12^ positive LN,^9^, ^10^, ^11^ hemoglobin level,^9^, ^10^ age,^4^, ^9^, ^10^, ^13^ performance status,^10^ positive surgical margin,^10^ tumor downstaging following NAC,^14^, ^15^, ^16^, ^17^ lactate dehydrogenase,^18^ presence of high‐grade hydronephrosis,^19^, ^20^, ^21^ preoperative renal function,^22^ SCC histology,^23^ systematic immune‐inflammation^9^, ^24^) on RFS, CSS, or OS. However, in addition to these well‐known risk factors, survival outcomes are influenced by unknown underlying multiple congenital and acquired risk factors. Therefore, individualized predictive models should be developed using neural network analyses based on all available clinicopathological factors and individual genetic information features.

However, in current real‐world clinical practice, it is desirable to make a risk‐stratified model or nomogram that can predict individual outcomes using available clinicopathological factors. Furthermore, it may be ideal to make reliable risk‐stratified models to predict all important survival outcomes (RFS, CSS, and OS) because each prediction model may have a different role in the clinical setting. For example, a reliable predictive model for RFS would be necessary to enable individualized follow‐up planning following radical cystectomy and to compare new surgical techniques and new neoadjuvant and adjuvant systematic treatment regimens, including new precision medicine against standard treatment strategies within a relatively short‐term. A reliable predictive model for both CSS and OS would be significant to inform more individualized prognoses to patients and relatives, to offer clinical studies for patients with predicted poor survival outcomes, and to compare lifelong survival outcomes for new perioperative combination therapies and new salvage treatment regimens against standard historical treatment strategies.

The COBRA score is a well‐known predictive model for estimating CSS by age, clinical T stage, and LN density using big data from the Surveillance, Epidemiology, and End Results (SEER) database.^4^ Variables in the COBRA score included pre‐ and postoperative clinical factors. However, initial variables of interest incorporated in a Cox proportional hazards model for COBRA score were only age, ethnic background, sex, grade, clinical tumor stage, and LN density because of the limitations in using a large database. In contrast, we could extract many available pre‐/intra‐/postoperative clinicopathological factors from our clinical records and revealed several independent predictive factors that significantly affected three distinct survival outcomes (RFS, CSS, and OS) using the Cox regression model by forward‐backward stepwise selection method. We objectively determined the risk assessment score for each independent predictive factor and developed a new reliable GUOSG scoring system predicting RFS, CSS, and OS (Tables 4 and 5).

From the viewpoint of further individualized risk stratification models, the original COBRA score can separate eight groups for CSS and is superior to the GUOSG score, which can separate three groups. However, previous external validation of the COBRA score using a large bi‐institutional cohort demonstrated the overlapping of several risk groups and asserted the use of the simplified model with three COBRA score risk categories.^25^ Furthermore, the COBRA study demonstrated OS in eight groups, but the performance of separating each COBRA score risk group may not be optimal, probably due to using the same three CSS predictive factors for OS.^4^ However, the GUOSG score for OS can separate five risk groups as very good (5‐year median OS; 100%), good (93.1%), average (75.7%), poor (65.1%), and extremely poor (15.4%), using specifically identified OS prognostic factors (age, clinical T, pathological N, tumor grade at ORC, and LVI). In contrast, the limitations in the present GUOSG prediction model may be the small number of datasets from a single institution. Remarkably, a recent phase 3 trial (SWOG S1011) revealed that extended LN dissection did not show any significant benefit on disease‐free survival or OS compared with standard LN dissection.^26^ Because our method for lymphadenectomy at ORC nearly conforms to standard LN dissection in the SWOG S1011 study, GUOSG scoring could be simply utilized for future external validation.

The low proportion (27.5%) of NAC in the present cohort might be a limitation of the GUOSG models. A relatively high proportion of patients (28.4% of the total cohort) undergoing ORC due to BCG refractory or multiple recurrent T1G3 bladder cancer may have resulted in a low proportion of NAC recipients in the total cohort. In contrast, 47.1% (24 of 51) patients with muscle‐invasive or LN‐positive bladder cancer received NAC since 2015, probably due to accumulated evidence.^27^ However, we have not obtained any conclusive answers as to who an appropriate candidate would be to undergo NAC before radical cystectomy in clinical settings. The previous landmark SWOG‐8710 study demonstrated that NAC utilizing M‐VAC increased median OS from 46 to 77 months, and the probability of having pT0 at radical cystectomy increased from 15% to 38%.^28^ A subsequent meta‐analysis also demonstrated that adding NAC using M‐VAC before radical cystectomy improved OS at an absolute 5.0%–6.5% benefit.^29^, ^30^ However, the efficacy of the M‐VAC regimen as NAC was lessened by unfavorable toxicity in a certain percentage of patients. In present clinical practice, the GC regimen is utilized in most patients because it has demonstrated an identical pathological response rate and OS to the M‐VAC regimen but with more tolerable toxicities. The probability of downstaging to pT0 in the present study was 23.2% (13/56) and 9.5% (14/148) in the NAC and non‐NAC groups, respectively, which were, respectively, comparable to the values of 38% and 15%, respectively, demonstrated in the previous study.^28^

Nonetheless, NAC for radical cystectomy candidates may have significant but modest benefits for OS, while it may have a risk of notable toxicity and delaying surgery for chemotherapy nonresponders. A survival advantage is observed mainly in NAC responders, and the response rate to pT0 for NAC in cT2 was higher than in T3/4 patients, at 39% and 24%, respectively.^31^ However, pT2 patients may not be appropriate candidates for NAC because there has been no randomized study to compare OS between pT2 patients treated with NAC and those without NAC. In general clinical practice, patients with apparent invasion to adjacent organs and surrounding fat tissue and pelvic LN swelling find it challenging to undergo immediate radical cystectomy and hence tend to undergo NAC. In the present cohort, NAC was recommended to patients with clinical node‐positive findings (Table S1), and undergoing NAC did not significantly affect RFS, CSS, and OS. It should be noted that, in univariate analyses, all three survival outcomes were significantly better in patients who exhibited downstaging to pT0 at ORC than those who did not, whereas downstaging to pT0 at ORC was not an independent predictive factor for any survival outcomes in the multivariate analyses. We cannot answer the clinical question on appropriate candidates for NAC in the present one‐arm study. However, the GUOSG model makes it possible to select unmet candidates with very poor RFS in the present standard of care, irrespective of NAC, to investigate the efficacy of newly developed systematic neoadjuvant regimens.

The database at our institution has not validated the GUOSG model because of the small sample size in the present cohort. However, several significant factors for predicting RFS, CSS, and OS could be identified by multivariate analyses, which may have covered the limitation on sample size by the time‐consuming elaborate forward‐backward stepwise selection method from univariate to multivariate analyses using almost all available clinicopathological factors. Nevertheless, conducting external validation on the GUOSG model worldwide is necessary.

In conclusion, the GUOSG model‐estimated RFS may have an essential role as a historical cohort while investigating the usefulness of adjuvant immune checkpoint inhibitors or new surgical techniques in the future. Furthermore, the estimated CSS and OS values by the GUOSG models may also have essential roles as historical cohorts in a future study investigating the usefulness of new salvage systematic treatment agents or maintenance treatment using immune checkpoint inhibitors in patients who have progressed to metastatic disease.

## AUTHOR CONTRIBUTIONS

**Akira Ohtsu:** Conceptualization (equal); data curation (equal); formal analysis (equal); writing – original draft (equal). **Seiji Arai:** Conceptualization (equal); data curation (equal); formal analysis (equal); writing – original draft (equal). **Yuji Fujizuka:** Data curation (equal). **Yoshiyuki Miyazawa:** Data curation (supporting). **Masashi Nomura:** Data curation (supporting). **Yoshitaka Sekine:** Data curation (supporting). **Hidekazu Koike:** Data curation (supporting). **Hiroshi Matsui:** Data curation (supporting). **Yasuhiro Shibata:** Conceptualization (equal); data curation (equal). **Kazuto Ito:** Conceptualization (equal); data curation (equal); formal analysis (equal); writing – original draft (equal). **Kazuhiro Suzuki:** Conceptualization (equal); supervision (equal).

## CONFLICT OF INTEREST STATEMENT

None of the authors have conflicts of interest that could be perceived as prejudicing the impartiality of the research reported, and none receive financial support from industrial companies related to this research.

## ETHICS STATEMENT

The protocol for this research project has been approved by the institutional review board (approval No. 1392) and ethics committee and conforms to the provisions of the Declaration of Helsinki.

## Supporting information


Tables S1–S3.

Table S4.

## Data Availability

The data supporting this study's findings are available from the corresponding authors (A.O. or S.A.) upon reasonable request.

## References

[1] Bladder cancer statistics . World Cancer Research Fund International. Accessed December 1st, 2022. https://www.wcrf.org/cancer‐trends/bladder‐cancer‐statistics/

[2] National Cancer Registry in Japan . National Cancer Registry. Cancer Statistics in Japan; 2016–2018. Accessed December 1st, 2022. https://ganjoho.jp/reg_stat/statistics/data/dl/en.html

[3] Cancer mortality from Vital Statistics in Japan . Cancer Statistics in Japan. 1958–2020. Accessed December 1st, 2022. https://ganjoho.jp/reg_stat/statistics/data/dl/en.html

[4] Welty CJ , Sanford TH , Wright JL , et al. The Cancer of the Bladder Risk Assessment (COBRA) score: estimating mortality after radical cystectomy. Cancer. 2017;123(23):4574‐4582. doi:10.1002/cncr.30918 28881475

[5] Palmieri F , Brunocilla E , Bertaccini A , et al. Prognostic value of lymphovascular invasion in bladder cancer in patients treated with radical cystectomy. Anticancer Res. 2010;30(7):2973‐2976. 20683041

[6] Park E , Ha HK , Chung MK . Prediction of prognosis after radical cystectomy for pathologic node‐negative bladder cancer. Int Urol Nephrol. 2011;43(4):1059‐1065. doi:10.1007/s11255-011-9920-2 21626133

[7] D'Souza AM , Pohar KS , Arif T , Geyer S , Zynger DL . Retrospective analysis of survival in muscle‐invasive bladder cancer: impact of pT classification, node status, lymphovascular invasion, and neoadjuvant chemotherapy. Virchows Arch. 2012;461(4):467‐474. doi:10.1007/s00428-012-1249-4 22915241

[8] Ku JH , Kang M , Kim HS , Jeong CW , Kwak C , Kim HH . Lymph node density as a prognostic variable in node‐positive bladder cancer: a meta‐analysis. BMC Cancer. 2015;15:447. doi:10.1186/s12885-015-1448-x 26027955 PMC4450458

[9] Zhang J , Zhou X , Ding H , et al. The prognostic value of routine preoperative blood parameters in muscle‐invasive bladder cancer. BMC Urol. 2020;20(1):31. doi:10.1186/s12894-020-00602-9 32192483 PMC7082918

[10] Soria F , Moschini M , Abufaraj M , et al. Preoperative anemia is associated with disease recurrence and progression in patients with non‐muscle‐invasive bladder cancer. Urol Oncol. 2017;35(3):113.e9‐113.e14. doi:10.1016/j.urolonc.2016.10.021 27908681

[11] Drakaki A , Pantuck A , Mhatre SK , et al. “Real‐world” outcomes and prognostic indicators among patients with high‐risk muscle‐invasive urothelial carcinoma. Urol Oncol. 2021;39(1):76.e15‐76.e22. doi:10.1016/j.urolonc.2020.07.011 32778476

[12] Jensen JB , Ulhøi BP , Jensen KM . Evaluation of different lymph node (LN) variables as prognostic markers in patients undergoing radical cystectomy and extended LN dissection to the level of the inferior mesenteric artery. BJU Int. 2012;109(3):388‐393. doi:10.1111/j.1464-410X.2011.10369.x 21851538

[13] Ferro M , Chiujdea S , Musi G , et al. Impact of age on outcomes of patients with pure carcinoma in situ of the bladder: multi‐institutional cohort analysis. Clin Genitourin Cancer. 2022;20(2):e166‐e172. doi:10.1016/j.clgc.2021.12.005 35033480

[14] Volkmer BG , Kuefer R , Bartsch G , et al. Effect of a pT0 cystectomy specimen without neoadjuvant therapy on survival. Cancer. 2005;104(11):2384‐2391. doi:10.1002/cncr.21475 16245327

[15] Teramukai S , Nishiyama H , Matsui Y , Ogawa O , Fukushima M . Evaluation for surrogacy of end points by using data from observational studies: tumor downstaging for evaluating neoadjuvant chemotherapy in invasive bladder cancer. Clin Cancer Res. 2006;12(1):139‐143. doi:10.1158/1078-0432.CCR-05-1598 16397035

[16] Kim DK , Kim JW , Jung HD , Ahn HK , Lee JY , Cho KS . Effects of adjuvant chemotherapy on locally advanced upper tract urothelial carcinoma: a systematic review and meta‐analysis. Clin Genitourin Cancer. 2019;17(6):e1193‐e1202. doi:10.1016/j.clgc.2019.08.010 31543442

[17] Zargar H , Espiritu PN , Fairey AS , et al. Multicenter assessment of neoadjuvant chemotherapy for muscle‐invasive bladder cancer. Eur Urol. 2015;67(2):241‐249. doi:10.1016/j.eururo.2014.09.007 25257030 PMC4840190

[18] Bhandari NR , Ounpraseuth ST , Kamel MH , et al. Changes in health‐related quality of life outcomes in older patients with kidney cancer: a longitudinal cohort analysis with matched controls. Urol Oncol. 2020;38(11):852.e811‐852.e820. doi:10.1016/j.urolonc.2020.08.015 32863123

[19] Kim DS , Cho KS , Lee YH , Cho NH , Oh YT , Hong SJ . High‐grade hydronephrosis predicts poor outcomes after radical cystectomy in patients with bladder cancer. J Korean Med Sci. 2010;25(3):369‐373. doi:10.3346/jkms.2010.25.3.369 20191034 PMC2826737

[20] Bartsch GC , Kuefer R , Gschwend JE , de Petriconi R , Hautmann RE , Volkmer BG . Hydronephrosis as a prognostic marker in bladder cancer in a cystectomy‐only series. Eur Urol. 2007;51(3):690‐697. doi:10.1016/j.eururo.2006.07.009 16904815

[21] Fernández MI , Williams SB , Willis DL , et al. Clinical risk stratification in patients with surgically resectable micropapillary bladder cancer. BJU Int. 2017;119(5):684‐691. doi:10.1111/bju.13689 27753185

[22] Koguchi D , Matsumoto K , Ikeda M , et al. Prognostic impact of preoperative renal function in patients treated with radical cystectomy: a multi‐institutional retrospective study. Int J Clin Oncol. 2020;25(11):1969‐1976. doi:10.1007/s10147-020-01745-3 32648134

[23] Matulay JT , Woldu SL , Lim A , et al. The impact of squamous histology on survival in patients with muscle‐invasive bladder cancer. Urol Oncol. 2019;37(6):353.e317‐353.e324. doi:10.1016/j.urolonc.2019.01.020 30704959

[24] Zhang W , Wang R , Ma W , et al. Systemic immune‐inflammation index predicts prognosis of bladder cancer patients after radical cystectomy. Ann Transl Med. 2019;7(18):431. doi:10.21037/atm.2019.09.02 31700867 PMC6803204

[25] Muilwijk T , Akand M , Soria F , et al. The Cancer of the Bladder Risk Assessment (COBRA) score for estimating cancer‐specific survival after radical cystectomy: external validation in a large bi‐institutional cohort. BJU Int. 2020;126(6):704‐714. doi:10.1111/bju.15163 32640103

[26] Lerner SP , Tangen C , Svatek RS , et al. SWOG S1011: a phase III surgical trial to evaluate the benefit of a standard versus an extended lymphadenectomy performed at time of radical cystectomy for muscle invasive urothelial cancer. J Clin Oncol. 2023;41:4508.

[27] Kitamura H , Tsukamoto T , Shibata T , et al. Randomised phase III study of neoadjuvant chemotherapy with methotrexate, doxorubicin, vinblastine and cisplatin followed by radical cystectomy compared with radical cystectomy alone for muscle‐invasive bladder cancer: Japan Clinical Oncology Group Study JCOG0209. Ann Oncol. 2014;25(6):1192‐1198. doi:10.1093/annonc/mdu126 24669010

[28] Grossman HB , Natale RB , Tangen CM , et al. Neoadjuvant chemotherapy plus cystectomy compared with cystectomy alone for locally advanced bladder cancer. N Engl J Med. 2003;349(9):859‐866. doi:10.1056/NEJMoa022148 12944571

[29] Collaboration ABCAM‐a . Neoadjuvant chemotherapy in invasive bladder cancer: update of a systematic review and meta‐analysis of individual patient data advanced bladder cancer (ABC) meta‐analysis collaboration. Eur Urol. 2005;48(2):202‐205. doi:10.1016/j.eururo.2005.04.006 15939524

[30] Winquist E , Kirchner TS , Segal R , Chin J , Lukka H , Genitourinary Cancer Disease Site Group CnCOPiE‐bCPGI . Neoadjuvant chemotherapy for transitional cell carcinoma of the bladder: a systematic review and meta‐analysis. J Urol. 2004;171(2 Pt 1):561‐569. doi:10.1097/01.ju.0000090967.08622.33 14713760

[31] Sonpavde G , Goldman BH , Speights VO , et al. Quality of pathologic response and surgery correlate with survival for patients with completely resected bladder cancer after neoadjuvant chemotherapy. Cancer. 2009;115(18):4104‐4109. doi:10.1002/cncr.24466 19517476 PMC3079554

